# Carrageenans, Sulphated Polysaccharides of Red Seaweeds, Differentially Affect *Arabidopsis thaliana* Resistance to *Trichoplusia ni* (Cabbage Looper)

**DOI:** 10.1371/journal.pone.0026834

**Published:** 2011-10-28

**Authors:** Jatinder S. Sangha, Wajahatullah Khan, Xiuhong Ji, Junzeng Zhang, Aaron A. S. Mills, Alan T. Critchley, Balakrishnan Prithiviraj

**Affiliations:** 1 Department of Environmental Sciences, Nova Scotia Agricultural College, Truro, Nova Scotia, Canada; 2 Department of Biochemistry, Genome Research Chair Unit, College of Science, King Saud University, Riyadh, Saudi Arabia; 3 Institute for Nutrisciences and Health, National Research Council of Canada, Charlottetown, Prince Edward Island, Canada; 4 Acadian Seaplants Limited, Dartmouth, Nova Scotia, Canada; French National Centre for Scientific Research, Université Paris-Sud, France

## Abstract

Carrageenans are a collective family of linear, sulphated galactans found in a number of commercially important species of marine red alga. These polysaccharides are known to elicit defense responses in plant and animals and possess anti-viral properties. We investigated the effect of foliar application of ι-, κ- and λ-carrageenans (representing various levels of sulphation) on *Arabidopsis thaliana* in resistance to the generalist insect *Trichoplusia ni* (cabbage looper) which is known to cause serious economic losses in crop plants. Plants treated with ι- and κ-carrageenan showed reduced leaf damage, whereas those treated with λ- carrageenan were similar to that of the control. In a no-choice test, larval weight was reduced by more than 20% in ι- and κ- carrageenan treatments, but unaffected by λ-carrageenan. In multiple choice tests, carrageenan treated plants attracted fewer *T. ni* larvae by the fourth day following infestation as compared to the control. The application of carrageenans did not affect oviposition behaviour of *T. ni*. Growth of *T. ni* feeding on an artificial diet amended with carrageenans was not different from that fed with untreated control diet. ι-carrageenan induced the expression of defense genes; *PR1*, *PDF1.2*, and *TI1*, but κ- and λ-carrageenans did not. Besides *PR1*, *PDF1.2*, and *TI1*, the indole glucosinolate biosynthesis genes *CYP79B2*, *CYP83B1* and glucosinolate hydrolysing QTL, *ESM1* were up-regulated by ι-carrageenan treatment at 48 h post infestation. Gas chromatography-mass spectrometry analysis of carrageenan treated leaves showed increased concentrations of both isothiocyanates and nitriles. Taken together, these results show that carrageenans have differential effects on *Arabidopsis* resistance to *T. ni* and that the degree of sulphation of the polysaccharide chain may well mediate this effect.

## Introduction

Plants have developed adaptive and dynamic responses to herbivores through defense mechanisms that are either constitutively expressed or induced following infestation. The induction of plant defenses allows the plant to execute responses with a high degree of specificity in a timely matter in order to maximize efficacy [1]. Furthermore, production of plant secondary metabolites can modulate insect performance by acting as toxins, repellents, or deterrents for generalists, whereas some compounds may act as guides for specialists, or mediate tri-trophic interactions [2], [3].

Inducible responses in plants commence through the rapid recognition of herbivores via the perception of elicitors that are present in the saliva of insects. These signal molecules trigger metabolic responses and induce the transcription of specific defense genes [4]–[8]. Besides elicitors of insect origin, a number of chemicals including oligo- and polysaccharides, peptides, proteins and lipids are also reported to elicit chemical responses that protect the plant from microbes and herbivorous insects [9], [10]. One novel source of plant defense elicitors is marine macroalgae [11]. Various algal polysaccharides, including laminarin (from brown seaweeds) and carrageenans, have the potential to induce disease resistance in plants and animals [12], [13]. Carrageenans are the major polysaccharide present in many red macroalgae (seaweed). These gel-forming polysaccharides have a linear backbone of D-galactose residues linked with alternating α-(1,3) and ß-(1,4) linkages which are substituted by one (κ-carrageenan), two (ι-carrageenan), or three (λ-carrageenan) ester-sulphonic groups per di-galactose repeating unit [14], [15]. The degree of sulphation of carrageenan molecules is believed to affect the induction of plant defense genes by triggering different metabolic pathways [12], [13]. Recent investigations have shown carrageenans to induce defens against various plant pathogens and mammalian viruses, however, the effect of carrageenans on plant resistance against insects is not known.

*Arabidopsis thaliana* is a model for studying plant insect interactions, host resistance mechanisms and induced plant defenses [16], [12]. *Arabidopsis* exhibit a high sensitivity to elicitors from insect, pathogen, or other chemicals such as methyl jasmonate (MJ), cis-jasmonate (CJ) and salicylic acid (SA) [5], [17]–[21]. Different elicitors may induce specific defense pathways and in some cases, overlapping responses are also reported. Interestingly, carrageenans have not been widely tested for plant defense responses but have been reported to strongly suppress certain mammalian viruses (including HPV and HIV) (http://www.freepatentsonline.com/y2010/0015247.html). However, it is not clear how structurally similar carrageenans elicit differential host responses. Since seaweed polysaccharides carrageenans, have been shown to induce plant defens responses against pathogens, it is plausible that carrageenans might induce resistance in plants against insect pests.

*Trichoplusia ni* (cabbage looper) (Lepidoptera: Noctuidae) is a polyphagous herbivore that feeds on a number of plant species including *Arabidopsis* [22]. *T. ni* has variable responses to different *Arabidopsis* ecotypes and has shown particular sensitivity to plant glucosinolates, defense signaling pathways and proteinase inhibitors [22], [23]. In addition, *T. ni* has been reported to preferentially feed on nitrile-producing *Arabidopsis* ecotypes, while isothiocyanate-producing ecotypes deter feeding [24], [25]. As *T.ni* has been shown to have a compatibility with *Arabidopsis*, as well as sensitivities to certain plant defense compounds, it is an excellent candidate for studies measuring the endogenous induction of plant defense compounds with exogenously applied carrageenans.

The purpose of this study was to evaluate the effects of differently sulphated carrageenans as elicitors of *Arabidopsis* resistance to *T. ni*. The effects of carrageenans on *T. ni* was evaluated through the measurement of leaf mass consumed by *T. ni* larvae and larval development. The nature of the carrageenan-induced resistance was evaluated by measuring expression of genes known to affect *Arabidopsis* resistance against herbivorous insects.

## Results

### Plant Responses to *T. ni* with Carrageenan Treatment

**Figure 1:**
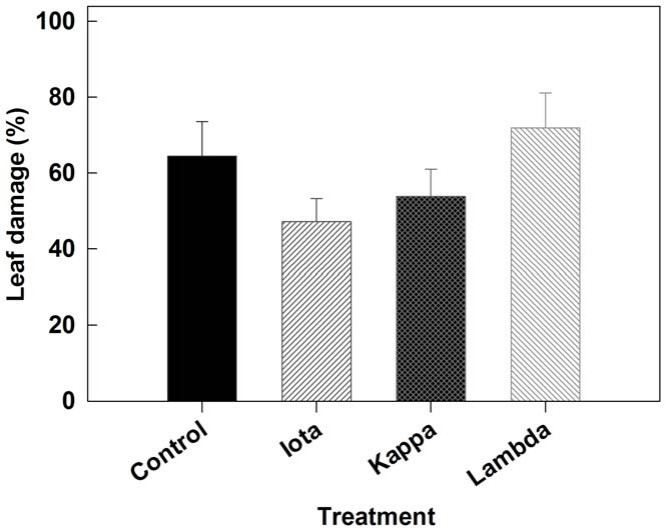
Leaf damage (%) on *Arabidopsis* treated with differentially sulphated ι- (iota), κ- (kappa) and λ- (lambda) carrageenans seven days following infestation. Plants with fully expanded leaves were sprayed until dripping with 2 ml of each test solution (1 g L^−1^) in ultra pure water (MilliQ) containing Tween-20 (0.02% v/v) followed by a second spray treatment on day five. The control plants were sprayed with sterile distilled water containing 0.02% Tween-20. Single newly hatched larvae were placed on the lower surface of an *Arabidopsis* leaf of treated plants that were kept individually in a plastic mesh cage under greenhouse conditions. Leaf damage was quantified 7 days after inoculation based on the total amount of leaf area consumed minus the initial area of the healthy leaf. Reduced mean leaf damage was observed in both the ι- and κ-carrageenan treatments. Error bars represent standard error.

*Arabidopsis* plants treated with ι-, κ- and λ- carrageenan showed differences in susceptibility to *T. ni* infestation compared to the untreated control plants. We quantified the total leaf damage at 7 days post infestation. Although differences were not statistically significant, observations of infested plants under no-choice conditions showed that the plants treated with ι- and κ-carrageenan incurred reduced feeding damage by *T. ni* larvae (Fig. 1). In contrast, plants treated with water (control) or λ-carrageenan showed similar levels of leaf damage.

### Carrageenans Modulated *T. ni* Larval Growth

**Figure 2:**
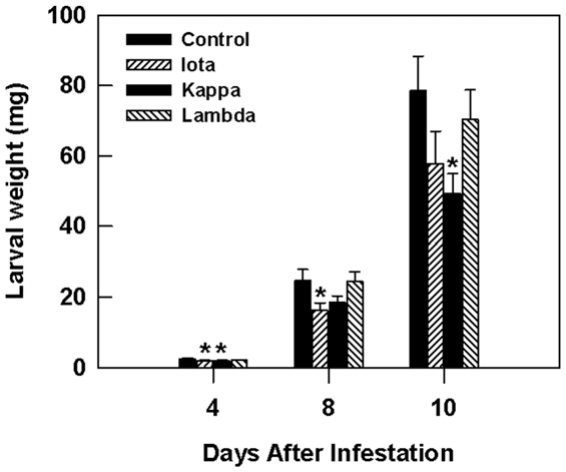
Sulphated carrageenans [ι- (iota), κ- (kappa) and λ- (lambda)] affect *T. ni* larval weight. Plants with fully expanded leaves were sprayed until dripping with 2 ml of each test solution (1 g L^−1^) in ultra pure water (MilliQ) containing Tween-20 (0.02% v/v) followed by a second spray treatment on day five. The control plants were sprayed with sterile distilled water containing 0.02% Tween-20. Single newly hatched larvae were placed on the lower surface of an *Arabidopsis* leaf of treated plants that were kept individually in a plastic mesh cage under greenhouse conditions. Larval fresh weight was measured 4, 8 and 10 days following infestation. The experiment was conducted under a randomized complete block design using five blocks consisting of five replicates each. Foliar application of carrageenans [ι- (iota), κ- (kappa)] reduced *T. ni* larval weight whereas λ- (lambda) did not. Error bars represent standard errors, and an asterisk (*) indicate significant differences (*p*<0.05) between mean values of the treatment (carrageenan) and the control (water).

The effect of carrageenan-treated *Arabidopsis* plants on *T. ni* larval growth was observed by measuring the larval fresh weight gain under confined feeding (no-choice) conditions. A reduction in larval weight was most obvious on the ι-carrageenan-treated plants and was significantly less (*p*<0.05) than the untreated control at 4, 8 and 10 d following infestation (Fig. 2). In κ-carrageenan-treated plants, the effect was not as pronounced as in the ι- carrageenan treated plants on days 4 and 8. However, by the tenth day following infestation, inhibition of larval growth was greater in the κ- than the ι- carrageenan-treated plants. In contrast, larval weight in the λ-carrageenan treatment was similar to that on the water control at all time points. Visual estimates of the size of the larvae feeding on ι-carrageenan-treated *Arabidopsis* was that they were smaller (Fig. S1), as well as an observed reduction in larval development as evidenced by delayed pupation compared to the control plants (data not shown).

### Larval development was unaffected on carrageenan amended artificial diet

**Figure 3:**
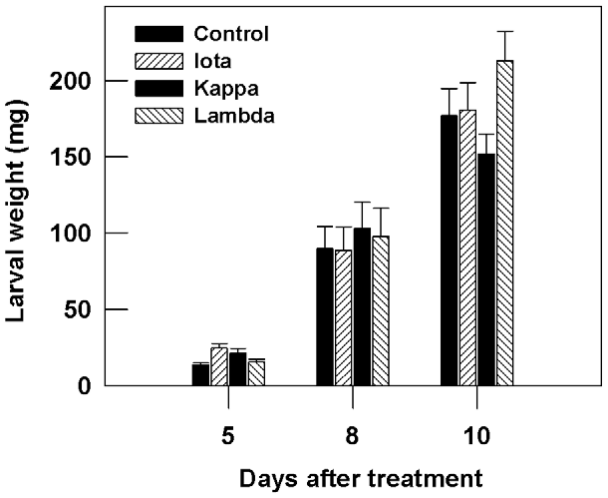
*T. ni* larval weight was unaffected on artificial diet laced with sulphated carrageenans [ι- (iota), κ- (kappa) and λ- (lambda)]. The artificial diet was prepared using dry diet ingredients of McMorran diet and carrageenans [ι- (iota), κ- (kappa) and λ- (lambda)] were added (1 g L^−1^) just before pouring into 5 ml plastic rearing cups. A single first instar larva was released on diet and incubated at 25°C. Larval weight was measured , 5, 8 and 10 days post infestation on the diet. The experiment was conducted under a randomized complete block design using three blocks consisting of fifteen replicates each. Error bars represent standard errors.

The direct effect of carrageenans on *T.ni* was observed by allowing *T. ni* larvae to feed on an artificial diet amended with carrageenans (1 g/L) and compared with normal (control) diet. The larval weight did not differ between the treatments at 5, 8 and 10 days post infestation (Fig. 3). The average larval period was 14.2 days for ι-carrageenan, 13.9 days for λ-carrageenan, 14.5 for κ-carrageenan and 14.3 days on the normal diet at 25°C.

### Carrageenan Treatment Affected *T. ni* Host Preference

**Figure 4:**
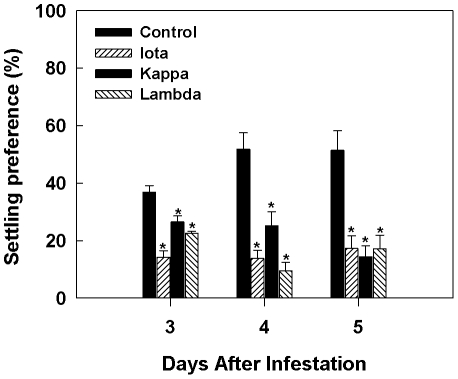
Sulphated carrageenans [ι- (iota), κ- (kappa) and λ- (lambda)] alter host preference of *T. ni*. *Arabidopsis* plants were sprayed with either carrageenans (1 g L^−1^) in ultra pure water (MilliQ) containing Tween-20 (0.02% v/v) or water and randomly arranged in a 25 cm circular tray at equal distance and placed in a mesh cage. Second instar *T. ni* larvae (5/plant) starved for one hour were placed in the center of the whorl and allowed to move freely to the host of their choice. Settling preference was determined by the numbers of larvae present on the treated plants at 3, 4 and 5 days following infestation. The experiment was repeated twice and consisted of five replicates per treatment. *T. ni* showed altered preference on carrageenans treated plants and least on **ι** -carrageenan sprayed plants. Error bars represent standard errors, and an asterisk (*) indicate significant differences (*p*<0.05) between mean values of the treatment (carrageenan) and the control (water).

Multiple choice experiments showed an overall reduction in *T. ni* larval preference of carrageenan- treated plants (Fig. 4). The larvae consistently showed non-preference for ι-carrageenan- treated plants. It was observed that the larval number did not change between plants up to 48 h after infestation. This trend however changed after 72 h and the *T. ni* larvae started moving away from the carrageenan-treated plants. The reduction in preference was greatest in the ι-carrageenan-treated plants on day three and it was followed by κ-carrageenan on day 4 and 5; the larval counts on λ-carrageenan treated plants showed similar trend on days 3 and 4. All carrageenan treatments showed significantly reduced *T. ni* preference by day five. By day 5 more than 50% of the larvae settled on the untreated control plants when given a choice (Fig. 4). Under dual choice conditions, although there was a reduction in larval settling preferences on ι- and κ-carrageenan-treated plants, the differences were not significant (data not shown). Conversely, there was increased settling preference in the dual choice experiment on plants treated with λ-carrageenan.

Female oviposition behaviour revealed that the oviposition behavior was not affected by carrageenan treatments. However, there were slight reduction in the number of eggs on the carrageenan-treated *Arabidopsis*, as compared to the control, but there were no significant differences (Fig. S2).

### Carrageenans Differentially Induce *Arabidopsis* Defense Genes

**Figure 5:**
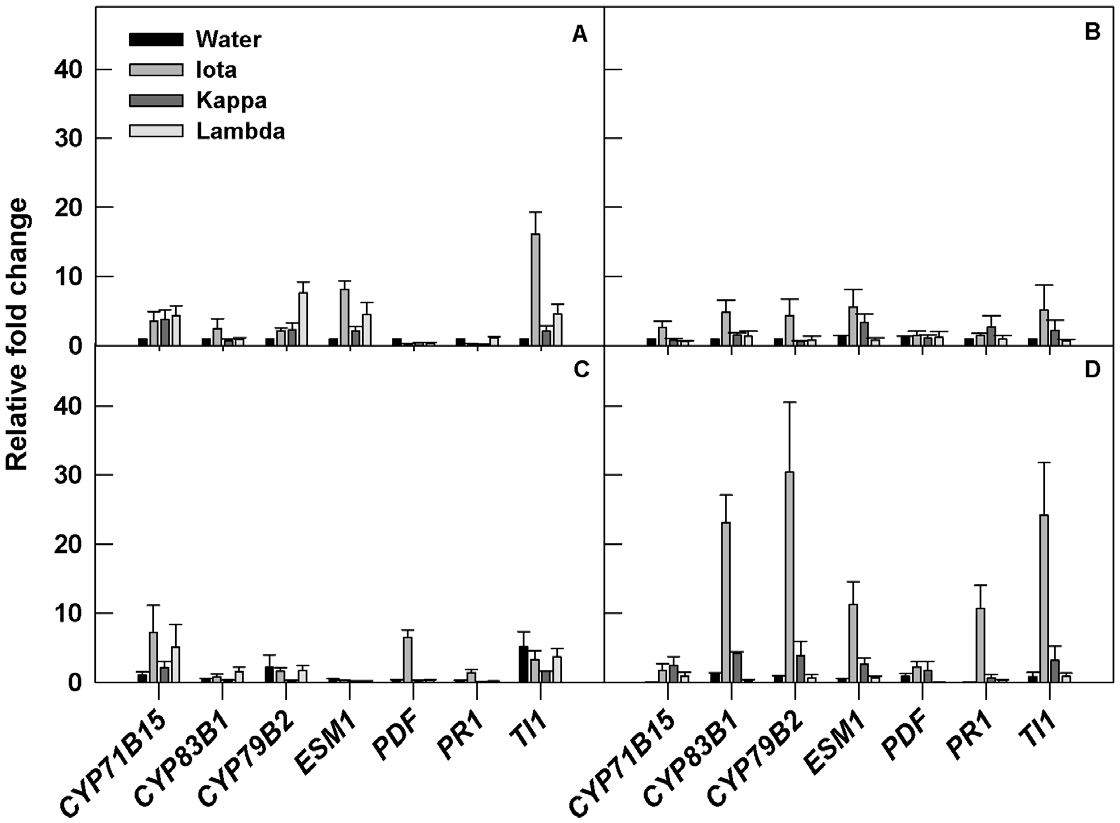
(A–D): Differential expression of defense genes against *T. ni* with sulfated carrageenans. Three week old plants were sprayed until dripping with 2 ml of each test solution (1 g L^−1^) in ultra pure water (MilliQ) containing Tween-20 (0.02% v/v) followed by a second spray treatment on day five. Pre-treated plants were infested with a single larva. At both 24 and 48 h following infestation, single leaves from five plants were harvested and pooled for RNA extraction. Relative gene expression of *CYP71B15*, *CYP79B2*, *CYP83B1*, and *ESM1 PR1*, *PDF1.2*, and *TI1* was determined with Real-time PCR performed on StepOne™ Real-Time PCR System using SYBR green dye with Rox (Roche). Data were analyzed from two independent Real-Time PCR runs. Transcript abundance of each selected gene is expressed relative to the expression in control healthy plants using the 2−^ΔΔCt^ method. Mean relative gene expression at (A) 24 h after application without *T. ni* infestation; (B) 48 hours after application without *T. ni* infestation (C) 24 hours after application with *T. ni* infestation; (D) 48 hours after application with *T. ni* infestation. Error bars represent SE of the mean of three independent runs.

To determine the molecular mechanisms of carrageenan-induced *Arabidopsis* defense to *T. ni*, marker genes commonly involved in *Arabidopsis* response to insect infestation were investigated. The relative expression of the genes *PDF1.2*, *PR1*, *TI1*, *CYP79B2*, *CYP71B15*, *CYP83B1*, *CYP81D11*, *OBP2* and *ESM1* were measured in carrageenan-treated plants up to 48 h post infestation (Fig. 5). Carrageenan treatments differentially induced the expression of these defense genes which concurred with phenotypic observations in the choice experiments. Jasmonic acid (PDF1.2) and salicylic acid-responsive (PR1) genes were induced by ι-carrageenan, but remained suppressed or unaltered during larval feeding on the κ- and λ-carrageenan-treated plants. A considerable increase of *PDF1.2* and *PR1* expression were observed in ι-carrageenan-treated plants at 24 h and 48 h after infestation (Fig. 5). The expression of *PDF1.2* increased slightly in κ-carrageenan treatments at 48 h after infestation. Both *PDF1.2* and *PR1* remained suppressed with *T. ni* infestation in λ-carrageenan-treated plants.

The transcript abundance of the trypsin inhibitor protein 1 (*TI1*), increased in healthy (control) plants 24 h after ι-carrageenan-treatment. However *TI1* was not altered in κ- and λ- treatments relative to the expression with ι-carrageenan (Fig. 5). Interestingly, the expression of *TI1* increased at 24 h after *T. ni* infestation in all the treatments except κ-carrageenan. This trend however changed at 48 h, as the transcript level of *TI1* increased several folds in ι-carrageenan-treated plants. The expression of *TI1* remained unchanged with the other carrageenan treatments similar to the response of control plants infested with *T. ni*.

We also determined whether sulphated carrageenans could alter glucosinolate biosynthesis products which might regulate defense of Arabidopsis to insects (Fig. 5). Indole glucosinolate biosynthesis genes *CYP79B2*, *CYP83B1* were differentially induced in carrageenan treatments during *T. ni* infestation. The induction of *CYP79B2* gene was observed at 24 h post carrageenan treatments without *T. ni* infestation and it was several folds higher with λ-carrageenan treatment. The induction of *CYP79B2*, increased further with ι-carrageenan but remained suppressed with other carrageenans at 48 h post treatment in absence of *T. ni* infestation. In contrast, *CYP79B2* remained suppressed at 24 h after infestation but dramatically increased in ι-carrageenan treatment 48 h after infestation. A four fold increase in *CYP79B2* transcript was also observed in κ-carrageenan treated plants.

The expression of *CYP83B1* showed >2 fold increase at 24 h after ι -carrageenan treatment but it was not induced in other treatments. Further, the expression of this gene which remained suppressed at 24 h after *T. ni* infestation was however increased >20 folds in ι-carrageenan and >4 folds in κ-carrageenan treated Arabidopsis at 48 h post infestation (Fig. 5). Similarly, the expression of Epithiospecifer Modifier 1 (*ESM1*), a gene involved in glucosinolate hydrolysis increased in healthy *Arabidopsis* plants after ι-carrageenan treatment. Similar to *TI1* response, *ESM1* was also suppressed initially with *T. ni* infestation at 24 h (Fig. 5). However, the *ESM1* was up-regulated again in ι-carrageenan treatment which was also evident as a slight increase was observed with κ-carrageenan treatment at 48 h after infestation. In contrast, the expression of *ESM1* did not increase with λ-carrageenan application during *T.ni* infestation, and the observed response was similar to the control plants.

We noted that the transcript of *CYP71B15* encoding a cytochrome P450 monooxygenase contributing towards camelexin biosynthesis was also altered after treatment with carrageenans than in untreated control plants. In *T.ni* infested plants at 24 h, *CYP71B15* expression was reduced in κ-carrageenan treatments, whereas the expression level remained higher in ι- and λ-carrageenan treated plants. However, *CYP71B15* expression was lower at 48 h as compared to 24 h. Additionally, we also determined the response of *CYP81D11* and *OBP2* genes in *T. ni* infested plants treated with carrageenans (Fig. S3). The induction of *OBP2*, which regulates glucosinolate biosynthesis in *Arabidopsis*, was much more evident with λ-carrageenan treatment under *T. ni* infestation. The induction of *OBP2* in ι- and κ-carrageenan-treated plants was also observed but it merely differed from the control plants. In contrast, *CYP81D11* was found to be up-regulated in the *T. ni* infested plants with all of the carrageenan treatments similar to its induction in the control plants.

In addition, the transcript abundace of *CYP79B2*, *ESM1* and *CYP71B15* were detected in *T. ni* infested plants with RT-PCR at 1, 2, 3, and 5 days post infestation in control and ι-carrageenan treated plants (data not shown). The expression of *CYP79B2* increased with infestation until day 2 with ι-carrageenan but decreased at days 3 and 5. Similarly ESM1 increased with ι-carrageenan at 48 h post infestation but reduced on other days;the level of expression was higher than the control. In contrast, the expression of *CYP71B15* was more in ι-carrageenan treated plants on days 1, 2 and 3 post infestation whereas its expression was lower on day 5 as compared to control plants.

### Carrageenans increase glucosinolate hydrolysis products

**Figure 6:**
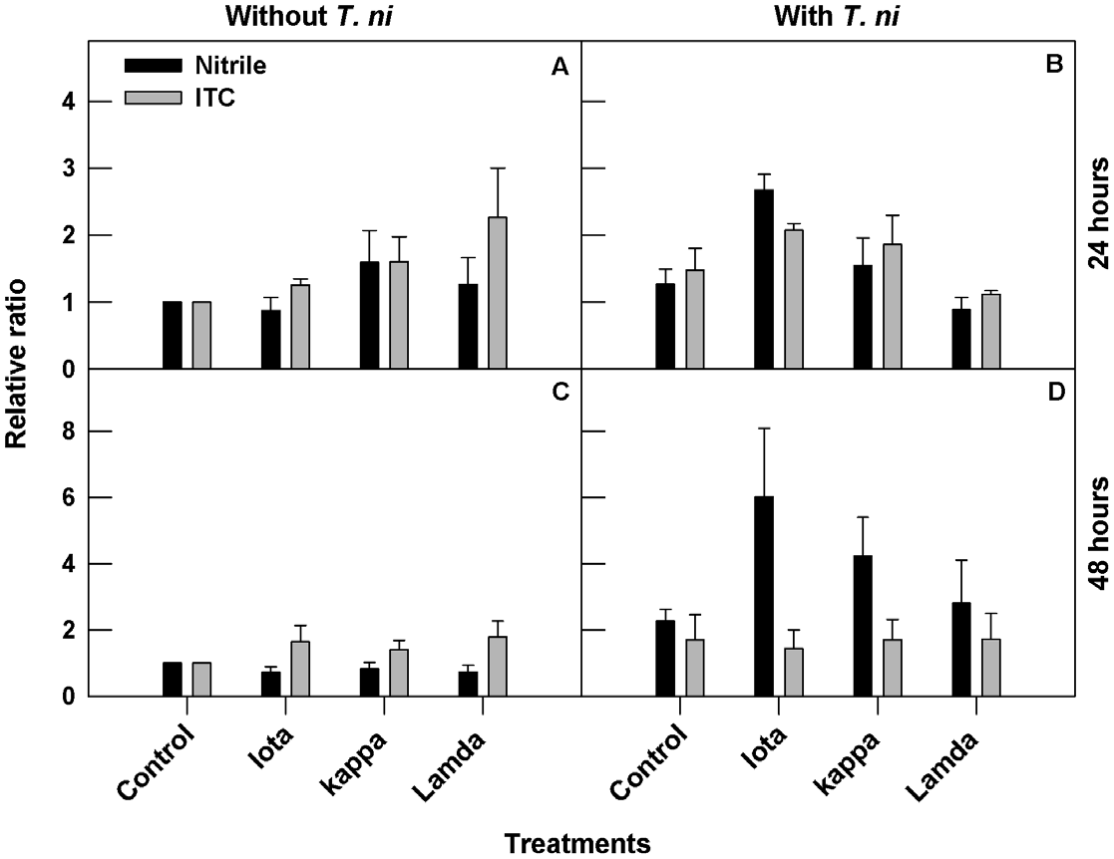
(A–D): Carrageenans modulate glucosinnolate hydrolysis products. Three week old plants were sprayed until dripping with 2 ml of each test solution (1 g L^−1^) in ultra pure water (MilliQ) containing Tween-20 (0.02% v/v) followed by a second spray treatment on day five. Pre-treated plants were infested with a single larva. At both 24 h and 48 h following infestation, six leaves were collected per three plants and used as replicate with and without *T. ni* for analysis of glucosinolate hydrolysis products analysis with GC-FID/EI MS according to Lambrix et al. [24]. The peak of 5.3 min is 3-pentenenitrile (NIT) and the peak at 7.6 min is L-sulforaphane [(-)-1-Isothiocyanato-(4R)-(methylsulfinyl)butane] (ITC). Data are expressed as the relative ratio of area under the absorbance peaks of a treatment to a water control. The experiment consisted of three biological replicates per treatment. Error bars represent SE of the mean of the replicates.

The glucosinolate profiles of carrageenan treated Arabidopsis plants were analyzed to determine if gene expression correlated with production of the toxic glucosinolate hydrolysis products, isothiocyanates and nitriles. Based on GC-FID analysis, the hydrolysis products showed distinct peaks with retention times of 5.3 and 7.6 min (Fig. S4 and S5). The peak of 5.3 min was identified as 3-pentenenitrile (NIT) based on NIST database of EI-MS. Whereas the peak at 7.6 min was L-sulforaphane [(-)-1-Isothiocyanato-(4R)-(methylsulfinyl)butane] (ITC) based on the analysis with standard (Sigma) and NIST database of EI-MS. We used relative ratio of area under absorbance peaks of a water treated control to differentiate the induction of hydrolysis products (Fig. 6, Figs. S4 and S5). The level of ITC was higher at 24 and 48 h post carrageenan treatments. Both ITC and NIT increased at 24 h post *T.ni* infestation and the concentration was higher with ι-carrageenan. However, we observed an increased NIT production at 48 h post infestation among all the carrageenans, whereas the levels of ITC were not different from the control (Fig. 6).

### ι-Carrageenan induced resistance requires JA and SA response

**Figure 7:**
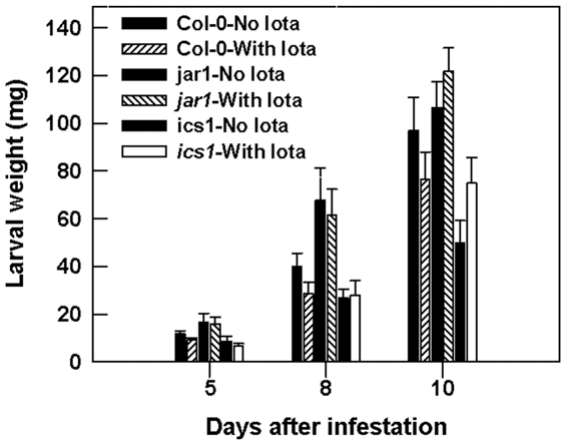
Arabidopsis mutants were not affected by ι-Carrageenan. Seedings of mutant plants (*ics1* and *jar1*) with fully expanded leaves were sprayed until dripping with 2 ml of ι-carrageenan solution (1 g L^−1^) in ultra pure water (MilliQ) containing Tween-20 (0.02% v/v) followed by a second spray treatment on day five. The control plants were sprayed with sterile distilled water containing 0.02% Tween-20. Single newly hatched larvae were placed on the lower surface of the plant leaf and kept individually in a plastic mesh cage under greenhouse conditions. Larval fresh weight was measured at 5, 8 and 10 days following infestation. The experiment was conducted under a randomized complete block design using three blocks consisting of five replicates each. Error bars represent standard errors of the mean.

We chose ι-carrageenan treatment to determine the response of the two Arabidopsis mutants *jar1* and *ics1*. ι-carrageenan did not affect the resistance of *jar1*, a mutant compromised in the JA dependent defense response, against *T. ni*. The larval weight on the ι-carrageenan-treated *jar1* plant was not significantly different (*p>*0.05) from that of the untreated control at 5, 8 and 10 days following infestation (Fig. 7). Similarly, larval weight on *ics1*, a mutant with a defect in SA biosynthesis, was not different from ι-carrageenan treated and untreated control plants at 5 and 8 days after infestation, although *T. ni* larvae showed a higher weight gain on ι-carrageenan treated plants on the 10^th^ day.

## Discussion

Seaweed extracts are increasingly used in agriculture to induce plant resistance to abiotic and biotic stresses. [26], [27]. Carrageenans specifically, have been reported to be potential inhibitors of fungal, bacterial and viral pathogens in plants and animals [11]–[13], [28]. This study demonstrated that spray treatment of carrageenans differentially modulated the resistance of *Arabidopsis* to *T. ni*, which is a generalist herbivore of several economically important food crops. Our results are in agreement with earlier published reports on the effect of elicitors such as jasmonic acid (JA), salicylic acid (SA), and 2,6-dichloroisonicotinic acid (INA) on inducing insect resistance in plants [17], [20].

The principal activity of ι-, κ- and λ-carrageenan is believed to be due to the number and position of the ester sulphate groups on the repeating galactose units which in turn may influence induction of defense in plants. λ-carrageenan has been shown to be the most active compound to elicit plant responses against plant pathogens [12], [13], [29], [30], [31], [32]. However in our study, insect response to carrageenan-treated plants and gene expression analyses indicated that the exogenous application of ι-carrageenan enhanced *Arabidopsis* resistance through antifeedant and antixenosis activities on *T. ni* larva. This observation is not surprising since the mechanisms of plant resistance to chewing insects are known to be different from that of pathogens. This finding was further supported by the experiments with direct feeding of carrageenans amended artificial diet bioassay which did not show any suppressive effects of carrageenans on *T. ni* growth and development. This effect could be in part due to the sulphation of ι-carrageenan, which is at an intermediate level to that of the κ- and λ- forms.

When attacked by insects, plants induce biochemical responses and alter the expression of defense related genes which can lead to the production of secondary metabolites and proteins including toxins, antifeedants, or anti-nutrients [33], [8]. We observed a higher expression of genes involved in the production of plant defense proteins, particularly in the ι-carrageenan-treated plants. Plant responses to chewing insects are largely JA dependent and this plays a major role in the activation of genes involved in induced defenses [17], [18], [34], [35]. It is interesting to note that both the JA and SA responsive genes *PDF1.2* and *PR1* were induced by ι-carrageenan application, with the induction of *PR1* being comparatively higher than *PDF1.2*. The response of two mutants *ics1* and *jar1* also suggested that both SA and JA dependent responses may be required in carrageenan induced Arabidopsis resistance against *T. ni*. “Cross-talk” between JA, SA and ET dependent defense pathways has been reported in plant defense mechanisms that interact with each other, and influence other pathways [8], [36], [37]. It seems likely that ι-carrageenan induced resistance involves multiple biochemical pathways in the development of plant resistance.

One of the most commonly induced herbivore defenses in plants is the rapid synthesis of anti-feedant proteinase inhibitors (PIs) [33], [38]. These small proteins inhibit insect digestive proteases and lead to reduced insect growth rates or their increased mortality due to a reduction of nutrient intake [39]. The several fold induction of *TI1* following ι-carrageenan treatment suggested that *TI1* probably played a major role in affecting the larval growth and development. This concept was supported by the observation of healthy larvae and more feeding damage on λ-carrageenan-treated plants which did not show an induction of the *TI1*. However, larval growth of *T.ni* was also suppressed with κ-carrageenan- treatment, although it did not correspond with strong *TI1* induction, thereby suggesting that implicit defense mechanisms were involved. The effect of κ-carrageenan on *T.ni* growth in both the choice and no-choice tests was intermediate to ι- and λ- carrageenan treatments of Arabidopsis.

In this study, carrageenan treatment of *Arabidopsis* induced several genes involved in indole glucosinolate biosynthesis including *CYP79B2* and *CYP83B1* that correlated with a pronounced phenotypic effect on *T. ni* larva. Interestingly, oviposition performance of gravid *T. ni* females was not affected with carrageenan treatment on Arabidopsis in choice experiment. This was surprising as *Arabidopsis* glucosinolates are defensive compounds which are involved in the production of volatiles, that have been shown to affect the performance of generalist herbivores such as *T. ni* through toxic isothiocyanates [22], [24], [40]. Conversely, specialist herbivores such as the cabbage butterfly redirects glucosinolate breakdown products toward less toxic nitriles with nitrile specific gut enzymes and use these compounds to locate host plants for feeding and oviposition [34], [41]. Since the glucosinolate biosynthesis gene activity was differentially induced in the treatments, it is possible that the glucosinolates or their by-products modulate carrageenan-induced resistance to *T.ni* larva but not the adults. However, λ-carrageenan-treated plants expressing higher *OBP2* and *CYP79B2* during the early stage of infestation appeared to incur more damage from larval feeding. It appeared that differential end products of glucosinolate biosynthesis may have mediated that response.

It is known that in the glucosinolate biosynthesis pathway, *ESM1* plays a pivotal role in the production of toxic glucosinolate by-products. *ESM1* is a semi-dominant quantitative trait locus (QTL) having an epistatic effect on the Epithiospecifier (ESP) gene [23]. It has already been determined that *T. ni* prefers to feed on nitrile-producing *Arabidopsis* ecotypes, whereas isothiocyanate-producing *Arabidopsis* mutants deter herbivores [24]. Up-regulation of *ESM1* represses nitrile formation and favours isothiocyanates which further deter *T. ni* herbivory [23]. Treatment of plants with ι-carrageenan, in this study, clearly led to increased *ESM1* which was associated with the production of isothiocyanates. This was also confirmed with GC-FID analysis for glucosinolate hydrolysis products. But *ESM1* expression did not correlate with a strong production of ITC at 48 h post-infestation. It is difficult to interpret this relationship due to the complex nature of the hydrolysis products linked with induced defense in Arabidopsis. In addition, *CYP71B15* involved in the final step of biosynthesis of antimicrobial camelexin could also contribute to carrageenan-induced Arabidopsis resistance to *T. ni*. This was evident from up-regulation of the *CYP71B15* gene transcript as observed at different times post-infestation.

**Figure 8:**
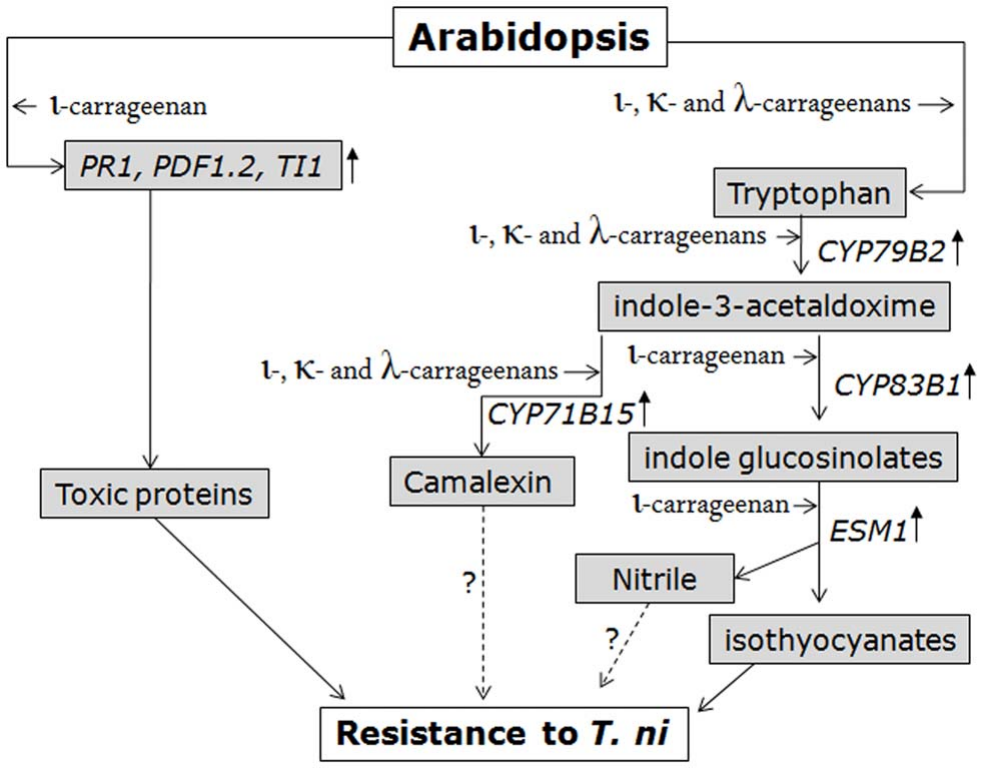
Schematic diagram of proposed carrageenan induced resistance in *Arabidopsis thaliana.* Genes regulating indole glucosinolates pathway are strongly upregulated with carrageenan treatments. The expression of genes, *CYP79B2* and *CYP83B1* increased with ι-carrageenan treatment. Finally, ESM1, representing an enzyme involved in glucosinolate hydrolysis into toxic isothyocyanates was up regulated with ι-carrageenan. Together with toxic plant proteins of *PR1*, *PDF1.2 and TI1* and indole glucosinolate byproducts, ι-carrageenan induced Arabidopsis resistance to *T.ni* larval infestation.

In summary, the treatment of plants with various carrageenans modulated the resistance of *Arabidopsis* to *T. ni* herbivory. In particular, ι-carrageenan elicited resistance to *T. ni* in *Arabidopsis* most likely by inducing various defense mechanisms including JA and SA-dependent pathways, proteinase inhibitors and an alteration of the products of glucosinolate hydrolysis as shown in Fig. 8. Carrageenan- induced resistance was expressed, through increased antibiosis and a non-preference type of plant resistance mechanism. In general, induced resistance offers the prospect of pest management through the exploitation of plant/pest resistance mechanisms by potential manipulation of both the intensity and timing of induced responses. Chemical elicitors, such as JA, SA, and benzo(1,2,3)thiadiazole-7-carbothioic acid (benzothiazole, BTH) are novel approaches to pest management that holds great potential for the possibility of being explored in common and widespread agricultural practices [42]. However, the cost of currently available and commonly recommended elicitors is high, and in some cases environmental toxicity has also been reported [43]. Seaweed-derived carrageenans hold promise as low-cost alternatives to chemically synthesized plant defense eliciting compounds. The red seaweeds *Chondrus crispus*, and various strains of *Eucheuma denticulatum* and *Kappaphycus alvarezii* contain a high percentage of defined types of carrageenan [44]. These naturally sourced carrageenans may be produced with relatively low cost, using simple methods and few negative environmental impacts have been reported for these polysaccharides. The information provided in the current study makes a case for future larger-scale experiments to measure the field efficacy of various types of carrageenans applied to plants against agricultural pests. Future studies on the role of carrageenan polysaccharides in modulating insect-plant interactions will be useful for pest control strategies and management which rely on induced plant resistance to herbivory.

## Materials and Methods

### Insect and Plant Material

*Trichoplusia ni* eggs were obtained from Agriculture and Agri-food Canada, Saskatoon, Saskatchewan, Canada. Before the initiation of the experiments, eggs were surface sterilized with sodium thiosulphate and incubated at 25°C for three days. The larvae which hatched were used to develop an in-house culture of *T. ni* which was maintained on an artificial diet (BioServ, Frenchtown, NJ) at 23±2°C, 65% relative humidity, and 16 h day and 8 h night cycles. Newly hatched larvae or gravid females were used for all greenhouse experiments.

Wild-type *Arabidopsis thaliana* (Heyhn.) ecotype Columbia (Col-0) seeds were purchased from Lehle Seed Company (Roundrock, TX, USA). Arabidopsis mutants (*ics1* and *jar1*) were obtained from the Arabidopsis Biological Resource Center (ABRC, Ohio State University Columbus, OH, USA). Seeds were planted in sterile peat pellets (Jiffy Co., Shippegan, New Brunswick, Canada) and kept in trays at 22±2°C with a photoperiod of 16 h day and 8 h night cycle. Three-week-old plants were used in the experiments.

### Preparation of Carrageenan Treatments

The three types of pure carrageenans (ι -, κ - and λ -) used in this study were provided as a gift by Cargill Texturant Solutions (Baupte, France). The level of sulphation of each of the carrageenans, viz. in descending order is λ-; ι-; κ-. Carrageenans were dissolved in ultra-pure water (MilliQ) (0.1% w/v) containing 0.02% (v/v) of Tween-20 at a 1 g L^−1^ concentration.

### Plant Treatment and Infestation

Treatments were applied to the *Arabidopsis* plants twice before infestation. Plants with fully expanded leaves were sprayed until dripping with 2 ml of each test solution followed by a second spray treatment on day five. The control plants were sprayed with sterile distilled water containing 0.02% Tween-20 (v/v). For Arabidopsis mutant analysis, only ι-carrageenan treatment was compared with water sprayed (control) plants. All experiments were conducted under greenhouse conditions (22±2°C, 16 h day and 8 h dark cycles). Infestation was performed 48 h following the second spray.

### No-Choice Test

To determine the direct antibiosis effects of carrageenan-treated plants on *T.ni*, a no-choice experiment was conducted by confining a single larva on an individual treated plant. Newly hatched larvae were carefully placed on the lower surface of an Arabidopsis leaf subjected to either control or carrageenan treatment. The infested plants were kept individually in a plastic mesh cage and maintained under greenhouse conditions, as described above. Leaf damage was quantified 7 days post infestation. The damaged leaves from treated plants were digitally scanned and analyzed with Winfolia (Regent Instruments Inc. Canada). The average leaf area was measured using healthy plants of similar age,. Leaf damage was quantified based on the total amount of leaf area consumed minus the initial area of the healthy leaf.

Larval fresh weight was measured 4, 8 and 10 days following infestation. Plants that were completely devoured were replaced with fresh Arabidopsis plants with the same treatment until the larvae pupated. The total larval period, i.e. the time to adult emergence and percentage of insect mortality were recorded. Plant resistance was determined based on the relative reduced larval weight and the relative amount of leaf area consumed by the larvae. The experiment was conducted under a randomized complete block design using five blocks consisting of five replicates each.

### Choice Test

To determine the indirect effects of carrageenan treated plants on *T. ni*, a choice test was conducted measuring larval settlement and female egg laying preferences on treated or control plants. *Arabidopsis* plants were sprayed with either carrageenans or water and were randomly arranged in a 25 cm circular tray at equal distances and placed under a protective mesh cage. Second instar *T. ni* larvae were removed from the rearing diet before the experiment and starved for one hour. Each plant was infested with five larvae placed in the center of the whorl of Arabidopsis leaves and allowed to move freely to the host of their choice. Settling preference was determined by the numbers of larvae present on the treated plants at 3, 4 and 5 days following infestation. The experiment was repeated twice and consisted of five replicates per treatment.

The binary preference response of newly moulted first instar larvae was also measured on detached Arabidopsis leaves treated with carrageenans. Five larvae were released between a control and treated detached leaves on opposite sides of a Petri dish. The number of larvae settling on leaves was recorded after 24 h.

Preference for oviposition by adult female *T. ni* on the treated or control plants was also measured. Four-week-old, treated plants, two from each carrageenan treatment or the untreated control, were placed randomly in a 30 cm wide circular tray and placed in a mesh cage under greenhouse conditions, as described above. Two gravid females were released onto the enclosed plants and confined for two days. Female adults were offered 10% honey solution for feeding. After two days, the females were removed and the number of eggs on each plant recorded. The experiment was conducted twice with three replicates in a completely randomized design.

### Larval feeding on carrageenan laced diet

To determine the direct effects of carrageenans on *T. ni*, larval growth was observed on artificial diet (McMorran diet) [45] amended with carrageenan (1 g/L) and compared with normal (control) diet. Dry diet ingredients for *T. ni* were purchased from Insect Production and Quarantine Laboratories, Natural Resources, Canada (WWW.insect.glfc.cfs.nrcan.gc.ca). The carrageenan was added to the artificial diet before being poured into 5 mL plastic rearing cups which had meshed caps. A single first instar larva was released on diet and incubated at 25°C. Larval weight was recorded at 5, 8 and 10 days after releasing them on the diet. Finally, the pupae were weighed for each treatment. The experiment was repeated twice with 25 replications in each trial.

### RNA Extraction and Gene Expression Analysis

To determine if Arabidopsis defense genes (Table 1) were expressed with various carrageenan treatments following *T. ni* infestation, pre-treated plants were infested with a single larva. At both 24 h and 48 h following infestation, leaves from five plants were harvested and pooled for RNA extraction. Total RNA was extracted using a phenol-free method [46]. Total RNA was reverse transcribed into cDNA using a superscript cDNA kit (Roche, Mississauga, ON, Canada).

**Table 1:** Primers used in the present study.

Gene	Description	Gene Locus	Primer (F = forward; R = reverse)
*OBP2*	OBF BINDING PROTEIN 2	AT1G07640	F-GGTCAACGCTCAAAGTCCTA
			R-AGCTTGGTTGTTGCCATTAG
*TI1*	TRYPSIN INHIBITOR PROTEIN 1	AT2G43510	F-GAATACGGAGGTGATGTTGG
			R-AAGCATTTGACGTTACTGCC
*CYP71B15*	CYTOCHROME P450 MONOOXYGENASE	AT3G26830	F-TTCCTCTGTTTCCTCGTCCT
			R-GCCAGCGACTCCACCAATCCC
*CYP79B2*	CYTOCHROME P450 MONOOXYGENASE	AT4G39950	F-ATGATGGGAAGCTTCTTTGG
			R-TCGCCGGATATCACATCC
*CYP83B1*	CYTOCHROME P450 MONOOXYGENASE	AT4G31500	F-TCACGGCCATATCTACCAGC
			R-TGGACGTCATGACTGGAC
*CYP81D11*	CYTOCHROME P450 MONOOXYGENASE	AT3G28740	F-CGAGAAACGTGTGGAGAAAG
			R-GACATCGCCCATTCTAACG
*PR1*	PATHOGENESIS-RELATED PROTEIN 1	AT2G14610.1	F-ACATGTGGGTTAGCGAGAAG
			R-ACTTTGGCACATCCGAGTCT
*PDF1.2*	PLANT DEFENSIN 1.2	AT5G44420.1	F-TGCTGGGAAGACATAGTTGC
			R-TGGTGGAAGCACAGAAGTTG
*ESM1*	EPITHIOSPECIFER MODIFIER PROTEIN 1	AT3G14210	F-TCGTAGGATTGCGACAGG
			R-CCTGAGCCTTCTCTGTGTTG

Real-time PCR was performed on a StepOne™ Real-Time PCR System (Applied Biosystems, CA) using SYBR green dye with Rox (Roche) according to the manufacturer's instructions. Reaction mixtures contained 50 ng of cDNA template from each sample, 20 ng of each gene specific primer and 7.5 µL of SYBR green reagent in a final volume of 20 µL. Data were analyzed from two independent Real-Time PCR runs. Transcript abundance of each selected gene was normalized to the expression of 18S ribosomal RNA. The data were analyzed using the 2-^ΔΔCt^ method.

### GC-FID Analysis for detecting Glucosinolate Hydrolysis Products

Leaves were collected 24 and 48 hours after treatment with the carrageenans with and without *T. ni* for glucosinolate hydrolysis product analysis according to the method of Lambrix et al. [24]. For all samples, 150 mg leaf tissues were ground using glass rod in 1.2 mL of sterile water in glass vials. The vials were kept for 5 min at room temperature to allow the hydrolysis of the glucosinolates. This was followed by addition of 4 mL dichloromethane to stop the reaction. Following vortexing for 3 s, the samples were centrifuged at 1100× g for 15 min and the organic phase was collected in a glass tube using a glass Pasteur pipette. The samples were extracted one more time and the organic phase was dried by passing over a column containing 1 g of anhydrous sodium sulfate (Sigma). The dried organic phase was dissolved in 150 µL of acetonitrile for gas chromatography with flame ionization detector and electron impact mass spectrometry (GC-FID/EI MS) analysis.

To analyze the extracted glucosinolate hydrolysis products, Agilent 6890N Network GC system was used using a modified method of Lambrix et al. [24] with detector temperature of 250°C and inlet temperature of 250°C. The oven temperature was first set at 40°C, and changed after 2 min to 250°C gradually (15°C/min). The oven was set to a final temperature of 250°C for 14 min. One microliter of sample extract was injected with splitless inlet mode for the detection of the hydrolyzed products. An Agilent high-resolution HP-5MS gas chromatography column (length, 30 m; i.d., 0.25 mm; film, 0.25 µm) was utilized. The peaks were analyzed using ChemStation software.

### Data Analysis

Data for the no-choice and choice experiments were analyzed using the mixed procedure (PROC MIXED) of Statistical Analysis Software (SAS) (Version 9.2, SAS Institute Inc., Cary, NC, USA) with the restricted maximum likelihood option and repeated measures with a compound symmetry correlation structure [47]. The univariate procedure of SAS was used to test for normality of residuals and outliers. Residuals were plotted against predicted values to evaluate homogeneity of variance and independence of predicted and residual values. T-tests were used to analyze binary choice tests using PROC TTEST in SAS.

## Supporting Information

Figure S1 **Sulphated carrageenans differentially inhibit** ***T. ni*** **larval development under confined feeding** Plants after dual sprays of each test solution (1 g L^−1^) in ultra pure water (MilliQ) containing Tween-20 (0.02% v/v) or control (sterile distilled water containing 0.02% Tween-20) were infested with single newly hatched larvae. Larvae representing the treatment effect were photographed at 10 days following infestation. The ι- (iota), and κ- (kappa) carrageenan more strongly retarded the *T. ni* larval growth than the λ- (lambda) carrageenan. The representative size of *T. ni* larvae feeding on ι-carrageenan-treated plants were smaller, as compared to other treatments.
(TIF)

Figure S2 **Oviposition behavior of** ***T. ni*** **females on** ***Arabidopsis*** **treated with sulfated carrageenans.** Three-week-old plants were given dual sprays of each test solution [1 g L^−1^ of (iota), κ- (kappa) and λ- (lambda) carrageenan] in ultra pure water (MilliQ) containing Tween-20 (0.02% v/v) or control (sterile distilled water containing 0.02% Tween-20). Two Plants from each carrageenan treatment, or the untreated control, were placed randomly in a 30 cm wide circular tray and placed in a mesh cage under greenhouse conditions. Two gravid females were released onto the enclosed plants and confined for two days. Female adults were offered 10% honey solution for feeding. After two days females were removed and the number of eggs on each plant was recorded. The experiment was conducted twice with three replicates under a completely randomized design. No significant differences were observed between the carrageenan treatments or the control. Error bars represent the standard error of the mean.
(TIF)

Figure S3 **Expression of** ***CYP81D11*** **and** ***OBP2*** **with ι- (iota), κ- (kappa) and λ- (lambda) carrageenan treatments** Three week old plants were sprayed until dripping with 2 ml of each test solution (1 g L^−1^) in ultra pure water (MilliQ) containing Tween-20 (0.02% v/v) followed by a second spray treatment on day five. Pretreated plants were infested with a single larva. At 24 h following infestation, infested leaves from five plants were harvested and pooled for RNA extraction. Relative gene expression *CYP81D11* and *OBP2* was determined with Real-time PCR performed on StepOne™ Real-Time PCR System using SYBR green dye with Rox (Roche). Data were analyzed from two independent Real-Time PCR runs. Transcript abundance of each selected gene was normalized to the expression of 18S ribosomal RNA. Mean relative gene expression of *CYP81D11* and *OBP2* at (A) 24 hours after application without *T. ni* infestation; (B) 24 hours after application with *T. ni* infestation. Error bars represent SE of the mean of two independent runs.
(TIF)

Figure S4 **GC-FID/EI MS peaks of glucosinolate hydrolysis products in carrageenan-treated plants.** Three week old plants were sprayed until dripping with 2 ml of each test solution (1 g L^−1^) in ultra pure water (MilliQ) containing Tween-20 (0.02% v/v) followed by a second spray treatment on day five. Pre-treated plants were infested with a single larva. At both 24 h following infestation, leaf samples were processed for extraction of glucosinolate hydrolysis products and subjected to GC-FID/EI MS analysis. The peak of 5.3 min was identified as 3-pentenenitrile (NIT) and the peak at 7.6 min as L-sulforaphane [(-)-1-Isothiocyanato-(4R)-(methylsulfinyl)butane] (ITC).
(TIF)

Figure S5 **GC-FID/EI MS peaks of glucosinolate hydrolysis products in carrageenan treated plants.** Three week old plants were sprayed until dripping with 2 ml of each test solution (1 g L^−1^) in ultra pure water (MilliQ) containing Tween-20 (0.02% v/v) followed by a second spray treatment on day five. Pre-treated plants were infested with a single larva. At 48 h following infestation, leaf samples were processed for extraction of glucosinolate hydrolysis products and subjected to GC-FID/EI MS analysis. The peak of 5.3 min was identified as 3-pentenenitrile (NIT) and the peak at 7.6 min as L-sulforaphane [(-)-1-Isothiocyanato-(4R)-(methylsulfinyl)butane] (ITC).
(TIF)

## References

[1] Agrawal AA (1999). Induced responses to herbivory in wild radish: effects on several herbivores and plant fitness.. Ecol.

[2] Kliebenstein DJ, Rowe HC, Denby KJ (2005). Secondary metabolites influence *Arabidopsis/Botrytis* interactions: variation in host production and pathogen sensitivity.. Plant J.

[3] Pieters CMJ, Dicke M (2007). Plant interactions with microbes and insects: from molecular mechanisms to ecology.. Trend Plant Sci.

[4] Cluzet S, Torregrosa C, Jacquet C, Lafitte C, Fournier J (2004). Gene expression profiling and protection of *Medicago truncatula* against a fungal infection in response to an elicitor from the green alga *Ulva* spp.. Plant Cell Environ.

[5] Kessler A, Baldwin IT (2001). Defensive function of herbivore-induced plant volatile emissions in nature.. Science.

[6] Kessler A, Baldwin IT (2002). Plant responses to insect herbivory: The emerging molecular analysis.. Ann Rev Plant Biol.

[7] Paré PW, Tumlinson JH (1999). Plant volatiles as a defense against insect herbivores.. Plant Physiol.

[8] Walling LL (2000). The myriad plant responses to herbivores.. J Plant Growth Regul.

[9] Alborn HT, Hansen TV, Jones TH, Bennett DC, Tumlinson JH (2007). Disulfooxy fatty acids from the American bird grasshopper *Schistocerca americana*, elicitors of plant volatiles.. Proc Natl Acad Sci U S A.

[10] Côté F, Ham KS, Hahn MG, Bergmann CW, Biswass BB, Das HK (1998). Oligosaccharide elicitors in host-pathogen interactions: generation, perception, and signal transduction.. Subcellular biochemistry: plant–microbe interactions, Vol 29.

[11] Khan W, Rayirath UP, Subramanian S, Mundaya NJ, Rayorath P (2009). Seaweed Extracts as Biostimulants of Plant Growth and Development.. J Plant Growth Regul.

[12] Mercier L, Lafitte C, Borderies G, Briand X, Esquerré-Tugayé MT (2001). The algal polysaccharide carrageenans can act as an elicitor of plant defense.. New Phytol.

[13] Sangha JS, Ravichandran S, Prithiviraj K, Critchley AT, Prithiviraj B (2010). Sulfated macroalgal polysaccharide ë-carrageenan induces resistance against *Sclerotinia sclerotiorum* in *Arabidopsis thaliana* whereas é-carrageenan enhances susceptibility.. Physiol Mol Plant Pathol.

[14] Gordon-Mills EM, McCandless EL (1975). Carrageenans in the cell walls of *Chondrus crispus* Stack. (Rhodophyceae, Gigartinales). I. Localization with fluorescent antibody.. Phycol.

[15] Zablackis E, Vreeland V, Doboszewski B, Laetsch WM (1991). Differential localization of carrageenan gelling sequences in *Kappaphycus alvarezii* var. Tambalang (Rhodophyta) with FITC-conjugated carrageenan oligosaccharides.. J Phycol.

[16] Van Poecke RMP, Dicke M (2002). Induced parasitoid attraction by *Arabidopsis thaliana*: involvement of the octadecanoid and the salicylic acid pathway.. J Exp Bot.

[17] McConn M, Creelman RA, Bell E, Mullet JE, Browse J (1997). Jasmonate is essential for insect defense in Arabidopsis.. Proc Natl Acad Sci.

[18] Reymond P, Farmer EE (1998). Jasmonate and salicylate as global signals for defense gene expression.. Curr Opin Plant Biol.

[19] Pieterse CMJ, Van Loon LC (1999). Salicylic acid-independent plant defense pathways.. Trend Plant Sci.

[20] Stotz HU, Pittendrigh BR, Kroymann J, Weniger K, Fritsche J (2000). Induced plant defense responses against chewing insects. Ethylene signaling reduces resistance of *Arabidopsis* against Egyptian cotton worm but not diamondback moth.. Plant Physiol.

[21] Glazebrook J, Chen WJ, Estes B, Chang HS, Nawrath C (2003). Topology of the network integrating salicylate and jasmonate signal transduction derived from global expression phenotyping.. Plant J.

[22] Jander G, Cui J, Nhan B, Pierce NE, Ausubel FM (2001). The TASTY locus on Chromosome 1 of *Arabidopsis* affects feeding of the insect herbivore *Trichoplusia ni*.. Plant Physiol.

[23] Zhang ZY, Ober JA, Kliebenstein DJ (2006). The gene controlling the quantitative trait locus EPITHIOSPECIFIER MODIFIER1 alters glucosinolate hydrolysis and insect resistance in *Arabidopsis*.. Plant Cell.

[24] Lambrix VM, Reichelt M, Mitchell-Olds T, Kliebenstein DJ, Gershenzon J (2001). The *Arabidopsis* epithiospecifier protein promotes the hydrolysis of glucosinolates to nitriles and influences *Trichoplusia ni* herbivory.. Plant Cell.

[25] Burow M, Markert J, Gershenzon J, Wittstock U (2006). Comparative biochemical characterization of nitrile-forming proteins from plants and insects that alter myrosinase-catalysed hydrolysis of glucosinolates.. FEBS J.

[26] Craigie JS (2010). Seaweed extract stimuli in plant science and agriculture.. J Appl Phycol.

[27] Pangestuti R, Kim S (2011). Neuroprotective effects of marine algae.. Mar Drugs.

[28] Stiles J, Guptill-Yoran L, Moore GE, Pogranichniy RM (2008). Effects of ë-carrageenan on *in vitro* replication of feline herpes virus and on experimentally induced herpetic conjunctivitis in cats.. IOVS.

[29] Carlucci MJ, Ciancia M, Matulewicz MC, Cerezo AS, Damonte EB (2004). Protective effect of a natural carrageenan on genital herpes simplex virus infection in mice.. Antiviral Res.

[30] Nagorskaya VP, Reunov AV, Lapshina LA, Yermak IM, Barabanova AO (2008). Influence of kappa/beta-carrageenan from red alga *Tichocarpus crinitus* on development of local infection induced by Tobacco Mosaic Virus in Xanthi-nc tobacco leaves.. Biol Bull.

[31] Fidantsef AL, Stout MJ, Thaler JS, Duffey SS, Bostock RM (1999). Signal interactions in pathogen and insect attack: expression of lipoxygenase, proteinase inhibitor II, and pathogenesis-related protein P4 in the tomato, *Lycopersicon esculentum*.. Physiol Mol Plant Pathol.

[32] Zarate SI, Kempema LA, Walling LL (2006). Silverleaf whitefly induces salicylic acid defenses and suppresses effectual jasmonic acid defenses.. Plant Physiol.

[33] Karban R, Baldwin IT (1997). Mechanisms of induced responses.. Induced Responses to Herbivory.

[34] De Vos M, Kriksunov K, Jander G (2008). Indole-3-acetonitrile production from indole glucosinolates deters oviposition by *Pieris rapae* (white cabbage butterfly).. Plant Physiol.

[35] Bodenhausen N, Reymond P (2007). Signaling pathways controlling induced resistance to insect herbivores in *Arabidopsis*.. Mol Plant Microbe Interact.

[36] Penninckx IA, Thomma BP, Buchala A, Metraux JP, Broekaert WF (1998). Concomitant activation of jasmonate and ethylene response pathways is required for induction of a plant defensin gene in *Arabidopsis*.. Plant Cell.

[37] Narusaka Y, Narusaka M, Seki M, Umezawa T, Ishida J (2004). Crosstalk in the responses to abiotic and biotic stresses in *Arabidopsis*: Analysis of gene expression in cytochrome P450 gene superfamily by cDNA microarray.. Plant Mol Biol.

[38] Broadway RM, Duffey SS, Pearce G, Ryan CA (1986). Plant proteinase inhibitors: a defense against herbivorous insects?. Entomol Exper Appl.

[39] Ryan CA (1990). Proteinase inhibitors in plants: genes for improving defenses against insects and pathogens.. Phytopathol.

[40] Bruce TJA, Matthes MC, Chamberlain K, Woodcock CM, Mohib A (2008). cis-Jasmone induces *Arabidopsis* genes that affect the chemical ecology of multitrophic interactions with aphids and their parasitoids.. Proc Natl Acad Sci U S A.

[41] Bidart-Bouzat MG, Kliebenstein DJ (2008). Differential levels of insect herbivory in the field associated with genotypic variation in glucosinolates in *Arabidopsis thaliana*.. J Chem Ecol.

[42] Bostock RM, Karban R, Thaler JS, Weyman PD, Gilchrist D (2008). Signal interactions in induced resistance to pathogens and insect herbivores.. Eur J Plant Pathol.

[43] Holopainen JK, Heijari J, Nerg AM, Vuorinen M, Kainulainen P (2009). Potential for the use of exogenous chemical elicitors in disease and insect pest management of conifer seedling production.. Open For Sci J.

[44] Chopin T, Sharp G, Belyea E, Semple R, Jones D (1999). Open-water aquaculture of the red alga *Chondrus crispus* in Prince Edward Island, Canada.. Hydrobiologia.

[45] McMorran A (1965). A synthetic diet for the spruce budworm, *Choristoneura fumiferana* (Clem.) (Lepidoptera: Tortricidae).. Can Entomol.

[46] Sangha JS, Gu K, Kaur J, Zhongchao Y (2010). An improved method for RNA isolation and cDNA library construction from immature seeds of *Jatropha curcas* L.. BMC Res Notes.

[47] Bowley SR (2008). A Hitchhiker's Guide to Statistics in Plant Biology 2^nd^ Ed..

