# Supplementary figures and images for: Carrageenans, Sulphated Polysaccharides of Red Seaweeds, Differentially Affect Arabidopsis thaliana Resistance to Trichoplusia ni (Cabbage Looper)
Source: PLoS One. 2011 Oct 28;6(10):e26834. doi: 10.1371/journal.pone.0026834 (PMC3203909; doi:10.1371/journal.pone.0026834)

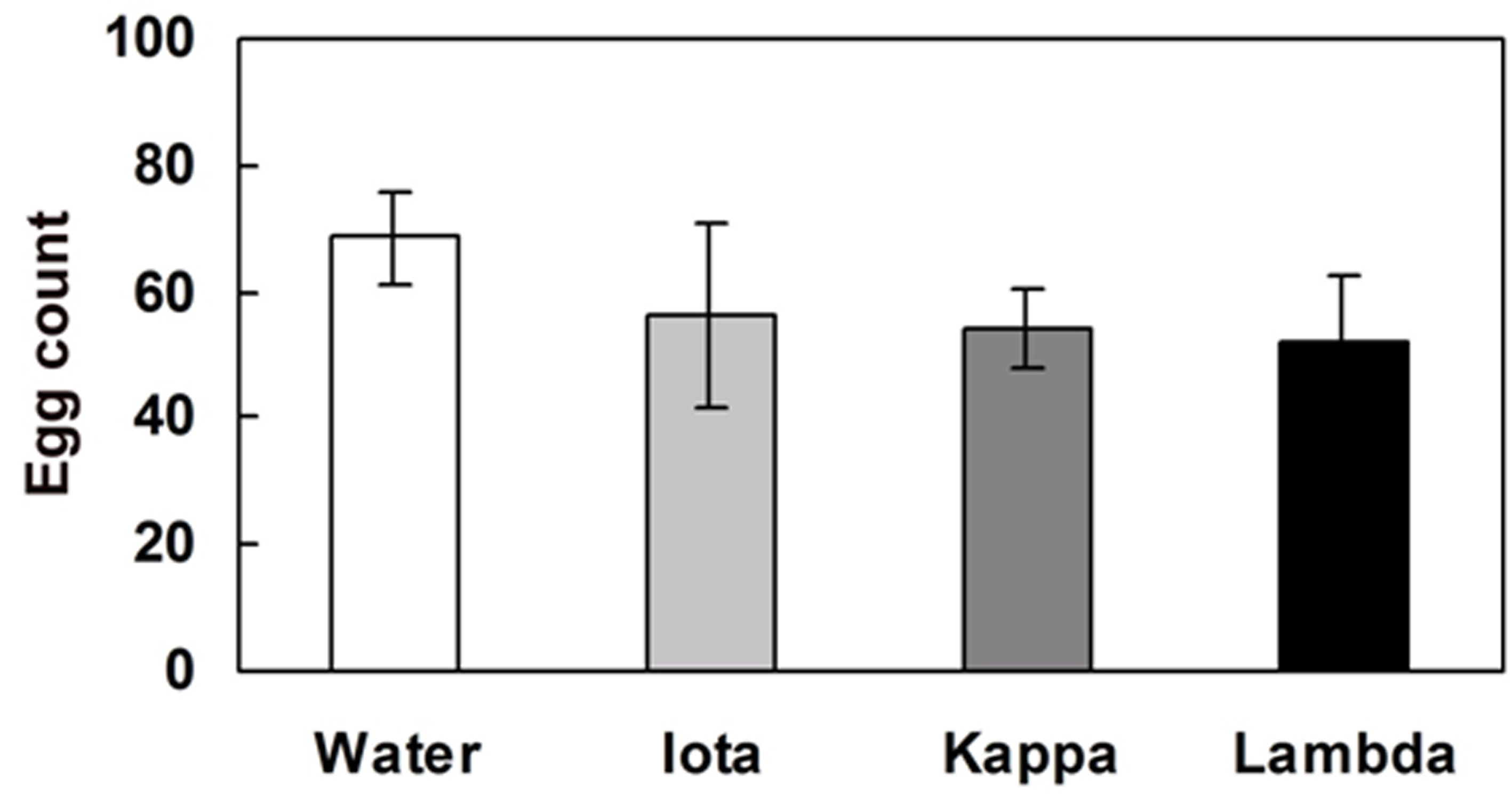

Supplement: Figure S2 — Oviposition behavior of T. ni females on Arabidopsis treated with sulfated carrageenans. Three-week-old plants were given dual sprays of each test solution [1 g L−1 of (iota), κ- (kappa) and λ- (lambda) carrageenan] in ultra pure water (MilliQ) containing Tween-20 (0.02% v/v) or control (sterile distilled water containing 0.02% Tween-20). Two Plants from each carrageenan treatment, or the untreated control, were placed randomly in a 30 cm wide circular tray and placed in a mesh cage under greenhouse conditions. Two gravid females were released onto the enclosed plants and confined for two days. Female adults were offered 10% honey solution for feeding. After two days females were removed and the number of eggs on each plant was recorded. The experiment was conducted twice with three replicates under a completely randomized design. No significant differences were observed between the carrageenan treatments or the control. Error bars represent the standard error of the mean. (TIF) [file pone.0026834.s002.tif]

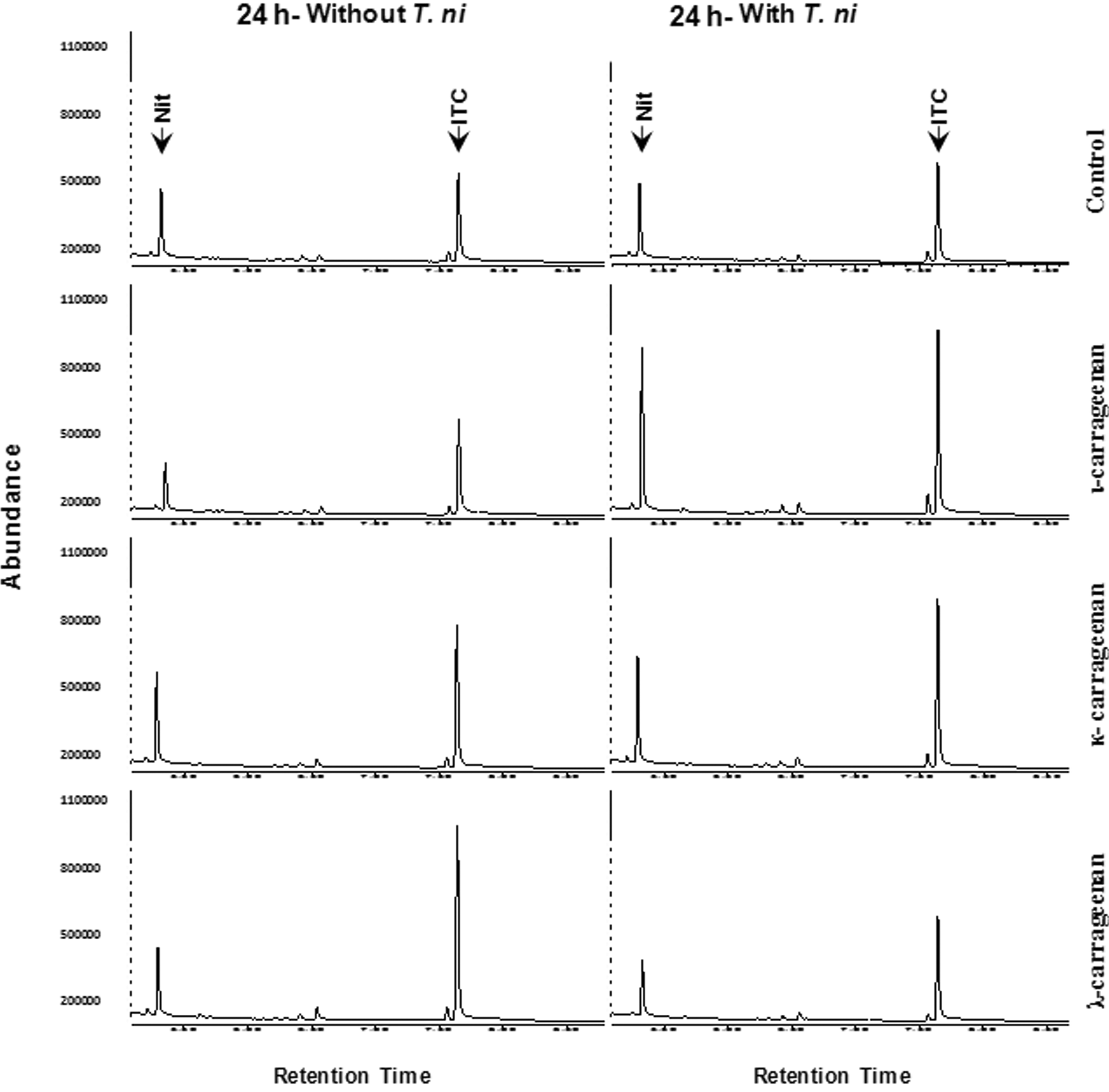

Supplement: Figure S4 — GC-FID/EI MS peaks of glucosinolate hydrolysis products in carrageenan-treated plants. Three week old plants were sprayed until dripping with 2 ml of each test solution (1 g L−1) in ultra pure water (MilliQ) containing Tween-20 (0.02% v/v) followed by a second spray treatment on day five. Pre-treated plants were infested with a single larva. At both 24 h following infestation, leaf samples were processed for extraction of glucosinolate hydrolysis products and subjected to GC-FID/EI MS analysis. The peak of 5.3 min was identified as 3-pentenenitrile (NIT) and the peak at 7.6 min as L-sulforaphane [(-)-1-Isothiocyanato-(4R)-(methylsulfinyl)butane] (ITC). (TIF) [file pone.0026834.s004.tif]

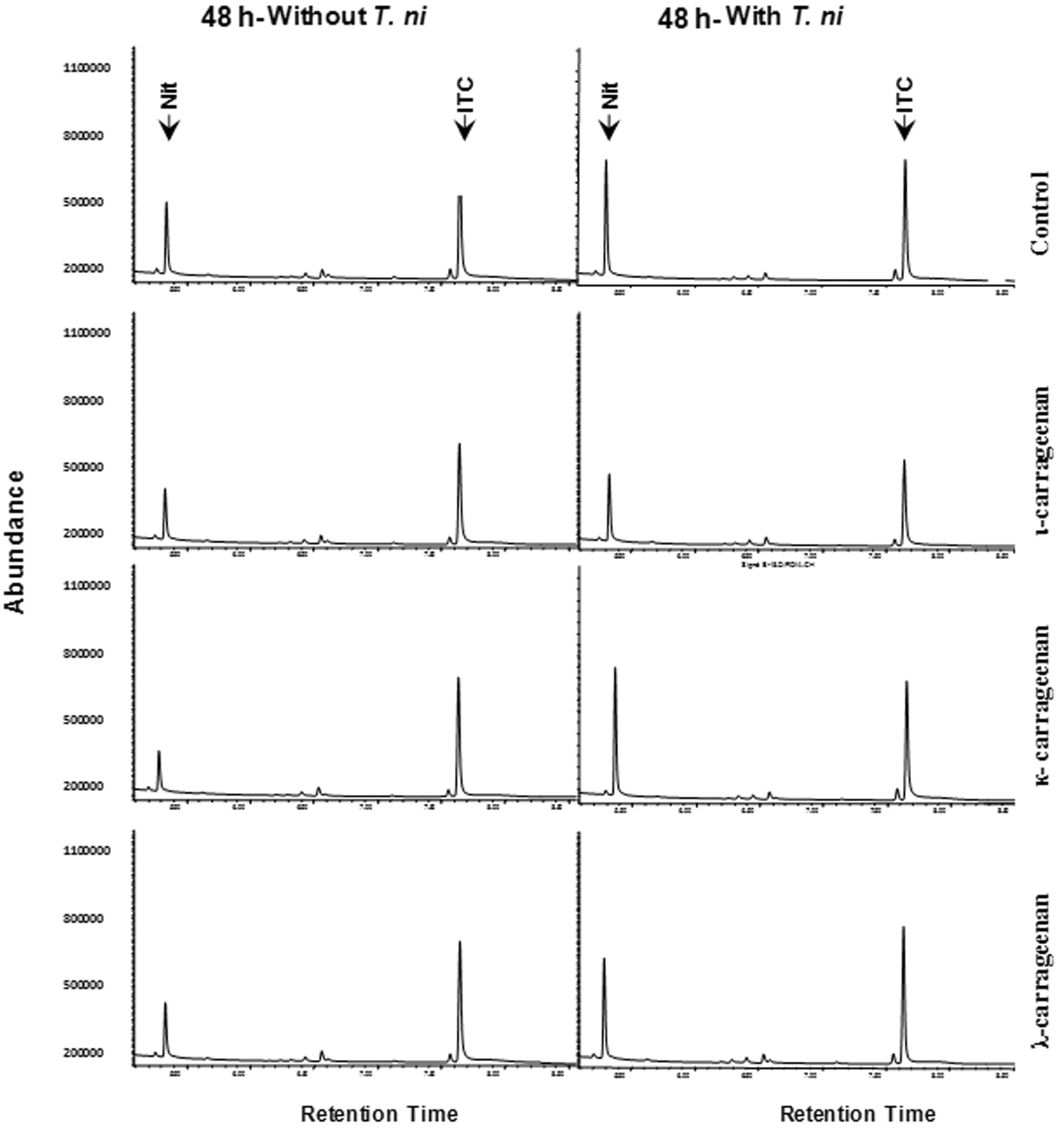

Supplement: Figure S5 — GC-FID/EI MS peaks of glucosinolate hydrolysis products in carrageenan treated plants. Three week old plants were sprayed until dripping with 2 ml of each test solution (1 g L−1) in ultra pure water (MilliQ) containing Tween-20 (0.02% v/v) followed by a second spray treatment on day five. Pre-treated plants were infested with a single larva. At 48 h following infestation, leaf samples were processed for extraction of glucosinolate hydrolysis products and subjected to GC-FID/EI MS analysis. The peak of 5.3 min was identified as 3-pentenenitrile (NIT) and the peak at 7.6 min as L-sulforaphane [(-)-1-Isothiocyanato-(4R)-(methylsulfinyl)butane] (ITC). (TIF) [file pone.0026834.s005.tif]
